# Healthcare consumption after a change in health insurance coverage: a French quasi-natural experiment

**DOI:** 10.1186/s13561-020-00275-y

**Published:** 2020-06-11

**Authors:** Christine Sevilla-Dedieu, Nathalie Billaudeau, Alain Paraponaris

**Affiliations:** 1 MGEN Foundation for Public Health, Paris, France; 2 Aix-Marseille Univ, CNRS, EHESS, Centrale Marseille, AMSE, Marseille, France; 3 ORS PACA, South-Eastern Health Observatory, Marseille, France

**Keywords:** Complementary health insurance, Moral hazard, Healthcare consumption, Longitudinal data, Exact matching, Difference-in-differences

## Abstract

**Background:**

Compared with the number of studies performed in the United States, few studies have been conducted on the link between health insurance and healthcare consumption in Europe, likely because most European countries have compulsory national health insurance (NHI) or a national health service (NHS). Recently, a major French private insurer, offering voluntary complementary coverage in addition to the compulsory NHI, replaced its single standard package with a range of offers from basic coverage (BC) to extended coverage (EC), providing a quasi-natural experiment to test theoretical assumptions about consumption patterns.

**Methods:**

Reimbursement claim data from 85,541 insurees were analysed from 2009 to 2018. Insurees who opted for EC were matched to those still covered by BC with similar characteristics. Difference-in-differences (DiD) models were used to compare both the monetary value and physical quantities of healthcare consumption before and after the change in coverage.

**Results:**

As expected, the DiD models revealed a strongly significant, though transitory (mainly during the first year), increase after the change in coverage for EC insurees, particularly for costly care such as dental prostheses and spectacles. Surprisingly, consumption seemed to precede the change in coverage, suggesting that one possible determinant of opting for more coverage may be previous unplanned expenses.

**Conclusion:**

Both catching-up behaviour and moral hazard are likely to play a role in the observed increase in healthcare consumption.

## Introduction

The literature on healthcare needs that have been unmet for financial reasons shows how much individual healthcare behaviours may be sensitive to monetary incentives [1, 2]. This finding may indicate a public health concern if the price affects how people produce health, mainly by accessing medical care that is important for long-term outcomes, which is especially important in chronic diseases such as diabetes or hypertension [3, 4], or how they take advantage of preventive medicine opportunities (screenings, vaccinations, dental check-ups) [5, 6]. This question, of course, is closely related to the issue of health insurance. A good example of this is the recent debate that took place in France as to whether the French national health insurance (NHI) should fully cover glasses, dental treatment, and hearing aids [7].

In contrast to the situation in the United States (US), where the literature on the link between health insurance and healthcare consumption is especially rich, following the RAND Health Insurance Experiment (HIE) [8] and the more recent Oregon Health Insurance Experiment (OHIE) [9], studies on this topic from Europe are not so common; where, why and how much to pay for health coverage are not the same. There are mainly studies on deductibles from Switzerland [10–12] and some others on recent variations in copayments, often for visits to primary care physicians, from Ireland [13], the Netherlands [14], Norway [15, 16], Portugal [17], Scotland [18], Spain [19, 20] and Sweden [21]. This lack of research is probably because in most European countries healthcare is either provided through the national health service (NHS), such as in the United Kingdom, or covered by compulsory standard health insurance, whether managed publicly (e.g., France, Germany) or by private companies (e.g., the Netherlands, Switzerland). Nonetheless, voluntary complementary health insurance (CHI) may coexist with the NHI, most often to supplement reimbursements from the NHI, when the NHI does not cover the entire cost. This may somewhat distort the results found in the US, showing that benefiting from health insurance increases the probability of seeking care [8, 10, 22, 23], the frequency of care [22, 24–27], and the extent of healthcare expenditure [8, 23, 24, 26, 28].

This is notably the case in France, where the NHI covers almost 100% of the population but is not fully comprehensive and financed only 77.8% of total healthcare expenditure in 2017 [29]. For this reason, more than 90% of the French population also chooses to subscribe to a CHI to cover the shortfall [30]. To date, a small number of French studies on the relationship between CHI and healthcare consumption [31–34] tend to confirm the overall findings from the US studies discussed above. However, most of these studies have analysed the impact of very small changes in CHI coverage on healthcare consumption, and only one performed a temporal analysis over a relatively long period of 5 years [33].

In 2011, a major French CHI company decided to move from a single standard basic coverage (BC) option to a range of offers by introducing an additional level of extended coverage (EC). This quasi-natural experiment provides the opportunity to determine trends in healthcare consumption after an improvement in health insurance coverage as well as before and, in particular, to determine whether this impact is limited to the types of consumption that are better reimbursed with EC. Our study used longitudinal reimbursement claims data from the period of 2009 to 2018. We studied how healthcare consumption changed, in both physical units and monetary expenses, for those who opted for EC compared with insurees who decided not to modify their coverage (BC). To control for observable heterogeneity between the EC and BC groups, which may explain discrepancies in healthcare consumption, individuals in the EC group were matched with individuals in the BC group. Trends in consumption were explored using difference-in-differences (DiD) models, which estimate differences in healthcare consumption observed before and after change, controlling for observed and unobserved heterogeneity.

## Methods

### Sample

The Mutuelle Générale de l’Éducation Nationale (MGEN) is one of the largest private not-for-profit organisations offering voluntary CHI coverage in France. For many years, the organisation has proposed a single BC to the members of one of its complementary schemes *Efficience Santé*, which covered over 140,000 individuals in 2017. However, since January 1, 2011, its affiliates have been offered the possibility to opt for EC. BC and EC reimbursements top up the NHI reimbursements to limit the insuree’s out-of-pocket payments. The differences in health insurance benefits between both levels of coverage are described in Table 1. The monthly premium for this CHI depends on the level of coverage that was subscribed to and the insuree’s age; the price starts at 27 € for BC and 33 € for EC [35].

**Table 1:** Examples of healthcare expenses and reimbursements by type of care and level of coverage in euros^a^

Insurance benefits	Expense	Reimbursement^b^	
		BC	EC
Visits to GPs	23.0	22.0	22.0
Visits to specialists	55.0	36.8	41.0
Visits to osteopaths	100.0	30.0	40.0
Pharmacy	107.0	74.5	94.5
Biological analyses	35.0	34.0	34.0
Paramedics	16.1	16.1	16.1
Medical procedures^c^	80.0	51.3	66.7
Dental care^d^	23.0	23.0	23.0
Dental prostheses	1200.0	709.7	768.8
Vision	330.0	174.4	214.4
Hospital	1026.0	627.0	655.0

^a^Examples detailed on the MGEN website [35]; ^b^total amount refunded by both the NHI and the CHI; ^c^procedures performed by a physician in relation to diagnosis, treatment or surgery; ^d^including dental consultations. BC: basic coverage; EC: extended coverage; GP: general practitioner

For this study, we considered only insurees who subscribed personally to this CHI (subscribers) and excluded other household members (spouses or children) as beneficiaries. We identified 873 EC insurees whose healthcare consumption was observable at least two full years before and after their change in coverage, which always became effective on the 1st of January. Of these 873 insurees, 4, 301, 155, 132, 110, 87 and 84 switched from BC to EC in 2011, 2012, 2013, 2014, 2015, 2016 and 2017, respectively. These 873 EC insurees were first matched to 873 of the 84,668 BC insurees who never changed their coverage. In a second step, it was possible to identify 838 pairs of EC and BC insurees that satisfied the parallel trends assumption of the DiD model. Details on the matching technique are given below.

### Data

For each insuree, data were extracted from the MGEN database for the years 2009 to 2018. For each year, the available individual information concerned the insurees’ sociodemographic data, including gender, age, marital status, employment status and area of residence; reimbursement claims; and administrative data concerning the level and period of coverage as well as the list of their enrolled dependents. Unfortunately, the health status of the participants is unknown since the French legislation on health data forbids its possession and use by complementary health insurers. The annual healthcare consumption was calculated in both euros and quantity (number of contacts, inpatient and outpatient care and drugs prescribed and delivered). These data were paired with sociogeographical variables produced by national statistical agencies that could affect healthcare supply and demand, namely, the type of town (urban/rural degree) [36] and access to physicians (density of general practitioners – GPs – and of specialists) [37].

### Empirical strategy

The impact of a change in EC coverage on healthcare consumption was estimated using a combination of two statistical techniques; one technique matched EC insurees to similar BC insurees, and the other consisted of DiD models to compare healthcare consumption before and after a change in coverage for these matched pairs with the aim of quantifying the difference in healthcare consumption attributable to the modified EC level.

First, to compare cases and controls, the 873 EC insurees were matched with 873 BC insurees who had similar characteristics in terms of gender, age, marital status and enrolled children. In addition, to control for self-selection, as individuals at higher health risk were expected to be most likely to change to EC, EC and BC insurees were also paired using two proxies of health status derived from reimbursement claims: a history of any hospital care, i.e., any care received in hospital as a day patient or an inpatient, and the need for specialised care, i.e., any care provided by a medical specialist. One-to-one matching was performed: each EC insuree was matched to a BC insuree. Since ordinal variables were used for matching, the exact matching (EM) method was adopted. A sensitivity analysis was, however, carried out using different selection options (one or multiple matched controls, with or without replacement, etc.) and alternative matching procedures, such as propensity score matching (PSM) or Kernel. Finally, as the date of change varied depending on the EC insuree (2011, 2012, 2013, 2014, 2015, 2016 or 2017), a comparison of healthcare consumption for each matched pair of EC and BC insurees was performed for the same full-four-year period.

**Fig. 1:**
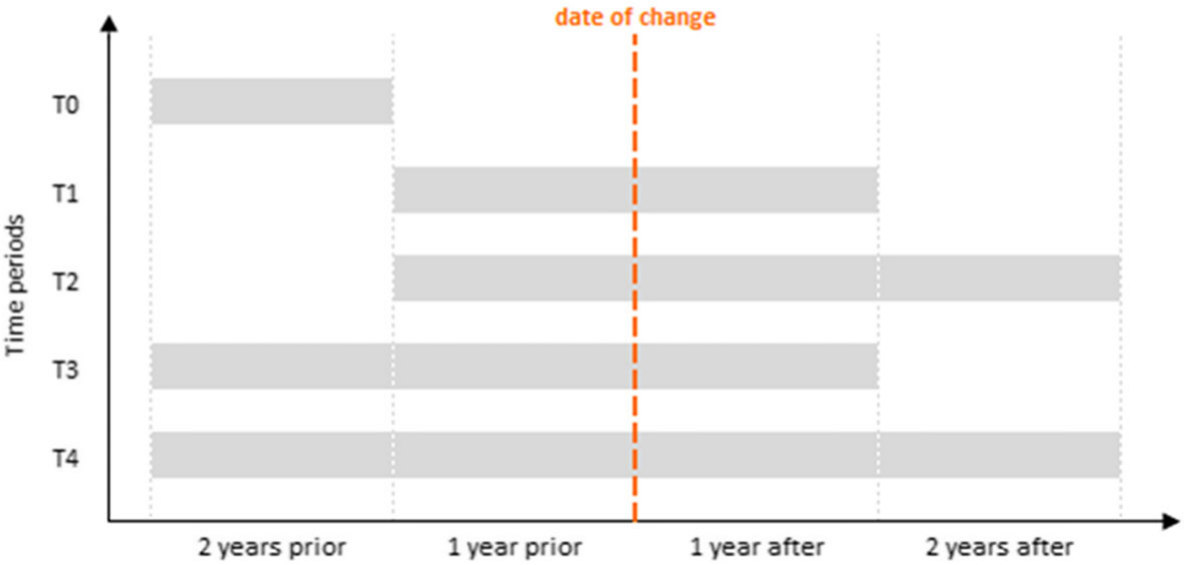
Time periods considered for the analysis

Second, the impact of CHI was estimated using DiD models, in which healthcare consumption was compared before and after a change in coverage. Different time periods were alternatively considered for the estimation (Fig. 1). A DiD design allows for the elimination of spurious effects due to secular trends in healthcare consumption (for example, a general increase in healthcare consumption) and unobserved factors that affect both the EC and BC groups (for instance, a policy reform affecting healthcare provision). The DiD estimates were also adjusted for all covariates presented in Table 2 to account for observed heterogeneity among insurees. The outcome variable was assessed both in current euros and in consumption units to control for potential distorting price effects.

**Table 2:** Characteristics of insurees in the year of change^a^

	Before EM				After EM			
	Total *N* = 85,541	Level of coverage			Total *N* = 1746	Level of coverage		
		BC *n* = 84,668	EC *n* = 873	p		BC *n* = 873	EC *n* = 873	*p*
Variable								
Female	49.0%	**48.9%**	**54.2%**	**.002**	54.2%	54.2%	54.2%	1.000
Age	40.6	**40.5**	**48.9**	**<.001**	48.9	48.8	48.9	.904
Living in couple	30.6%	**30.5%**	**37.5%**	**<.001**	37.5%	37.5%	37.5%	1.000
Any dependent child	18.2%	**18.3%**	**14.0%**	**.001**	14.0%	14.0%	14.0%	1.000
Dependent spouse	1.4%	**1.3%**	**3.0%**	**<.001**	2.7%	2.4%	3.0%	.460
Employed	70.1%	**70.2%**	**64.3%**	**<.001**	63.6%	62.9%	64.3%	.559
Any specialised care	38.5%	**38.3%**	**62.8%**	**<.001**	62.8%	62.8%	62.8%	1.000
Any hospital care	18.7%	**18.6%**	**30.0%**	**<.001**	30.0%	30.0%	30.0%	1.000
Urban location	86.2%	86.2%	85.0%	.313	84.7%	84.3%	85.0%	.692
Density of GPs^b^	104.0	103.9	107.2	.172	104.9	102.7	107.2	.147
Density of medical specialists^b^	87.5	87.5	90.0	.461	88.1	86.1	90.0	.408

Descriptive statistics include means for continuous variables and proportions for categorical variables. Statistical comparisons were performed between groups using Student’s *t*-test for means and the *z*-test for proportions. ^a^ For the BC controls, the date considered corresponds to the date of change in the EC pairs. ^b^ Per 100,000 inhabitants. A p-value less than 0.05 (in bold) indicates a significant difference between groups. EM: exact matching; BC: basic coverage; EC: extended coverage

**Fig. 2:**
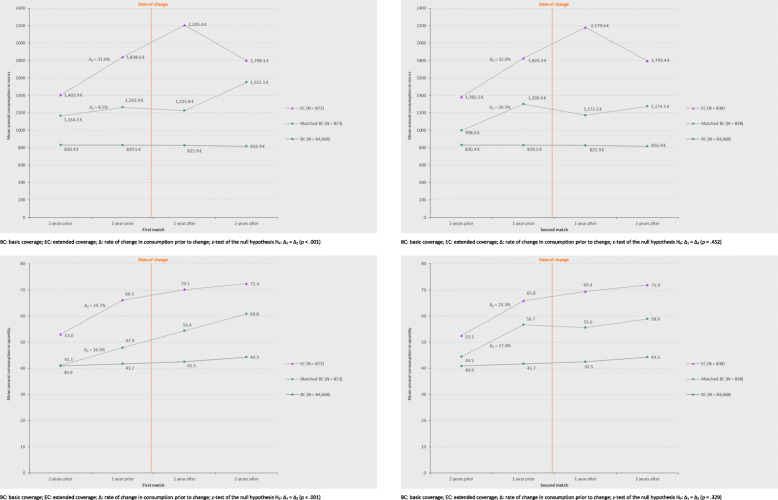
Average healthcare consumption in euros and quantity during the years before and after the change

The parallel trends assumption, which is the key assumption of DiD, assumes no difference in consumption trends between the EC and BC groups prior to the change in coverage. This assumption is often difficult to verify, but as we had 2 years of data before the change in coverage, we could compare the healthcare consumption between the EC and BC groups. As shown in Fig. 2, the first match did not satisfy the assumption, with rates of change in consumption between matched groups being significantly different before the change based on a *z*-test (*p* < .001 for both euros and physical units). Before estimating DiD, we thus identified alternative pairs of EC and BC insurees with comparable variation rates in consumption before change in both euros (*p* = .452) and quantity (*p* = .329) in addition to similar characteristics for the variables listed above. The second match satisfying these more restrictive conditions included slightly fewer pairs (838 insurees in each group). The DiD models were thus estimated with these new matched groups. Another common robustness check for this assumption is to estimate DiD for the pre-change period only. We thus estimated DiD models for the 2 years (T0 period) when all insurees had the same health insurance coverage since the EC group had not yet switched from the basic level to the EC (Fig. 1). Since the entire T0 period precedes the change in coverage (EC), the DiD estimator should not be significant if the assumption is valid. This alternative way of testing whether the pre-change consumption was different between the EC and BC groups is complementary to the previous one and allows this assumption to be tested both for overall healthcare and for specific categories of consumption.

## Results

### Characteristics of insurees

Table 2 compares the features of the EC and BC insurees. Before matching, we observed significant differences in all variables analysed, with the exception of those related to medical supply (*p* > .172), and urban location (*p* = .313). For example, in EC subjects there was a higher proportion of women (54.2% versus 48.9%; *p* = .002), insurees living as couples (37.5% versus 30.5%; *p* < .001), insurees having coverage for a spouse or a partner (3.0% versus 1.3%; *p* < .001) and insurees having been cared for by a specialist (62.8% versus 38.3%; *p* < .001) or in a hospital (30.0% versus 18.6%; *p* < .001). Conversely, EC subjects had a lower rate of employment (64.3% versus 70.2%; *p* < .001) and of insurees having coverage for any child (14.0% versus 18.3%; *p* = .001). Moreover, there was a strong difference between both groups with respect to age. Individuals were on average 48.9 years old in the EC group and 40.5 years in the BC group (*p* < .001). As expected, after matching, all of these differences were eliminated.

### Pattern of healthcare consumption

The insurees who opted for EC had a much higher level of consumption than the BC insurees regarding both euros and consumption units (Fig. 2), although the BC group increased their consumption over the period by 8% in physical units. For the matched BC insurees, the level of consumption was higher than that of all BC insurees but was still lower than that of the EC group.

In the EC group, healthcare consumption in euros began increasing at least 1 year before the change in insurance coverage. In the year following the change, consumption increased even more, by approximately 20% compared with the previous year, when it was at approximately 1840 €, to reach a maximum of more than 2200 € per insuree on average. However, 2 years after the change in coverage, consumption in euros returned to approximately the same level as in the year before the change, approximately 1800 €. Regarding consumption units, we observed the same increase 1 year before the change in coverage as for consumption in euros. However, after the change, the trend for the EC insurees was completely different, as consumption increased at a slower pace, 6% then 3% instead of 25% before extension. This divergence between consumption measured in euros and that measured in physical units after the change in coverage suggests that costly items were consumed immediately after the change.

When analysing the evolution of each category of care, it is worth noting that for the EC insurees expenditures substantially increased in the year before the change in the seven categories of care (Additional file 1), namely, hospital care (+ 95.5%), paramedical visits (+ 82.6%), dental prostheses (+ 54.0%), medical acts (+ 48.1%), dental care (+ 44.0%), specialised care (+ 34.2%) and vision (+ 29.8%). In the year after the change in coverage, the consumption of dental prostheses more than doubled (+ 146.7%), and the expenditure related to vision and biological analyses increased by approximately 30%. However, the peak in consumption for these three categories did not last for more than a year, returning 2 years later to approximately the level observed 1 year prior to the change. When consumption was calculated as healthcare units (Additional file 2), similar trends were generally observed, with a sharp increase 1 year after the change in coverage for dental prostheses (+ 100.0%), pharmacy (+ 25.1%), and vision (+ 22.2%) together with a substantial increase 1 year prior to the change in paramedical visits (+ 84.0%), hospital stays (+ 66.7%), biological analyses (+ 39.3%), dental care (+ 36.4%), specialised care (+ 34.6%), medical procedures (+ 29.6%), vision (+ 28.6%) and pharmacy (+ 28.2%).

### Assessment of the impact of health insurance on healthcare consumption using DiD models

Table 3 shows that differences in the patterns of healthcare consumption between the two groups varied considerably according to the time period considered (Fig. 1). As required by the parallel trends assumption, no significant difference in care consumption between the groups was observed upstream of the decision (T0), with the exception of dental care in euros only (*p* = .026). Regarding differences before and after making the decision to change coverage, a significant substantial increase in overall healthcare consumption in euros was found for different time intervals, T1 (*p* = .005), T3 (*p* < .001) and T4 (*p* = .046), whereas no effect was found in units. When considering categories of care, the most significant positive impacts were found for dental prostheses and to a much lesser extent for vision, both for consumption expressed in euros and as units. In addition, subscribing to better insurance coverage had a significant positive impact on the consumption of medical procedures and biological analyses in euros. It should be noted, however, that the size and significance of these differences were lower when measuring the impact 2 years later, suggesting that the increase in consumption after the change in coverage did not generally persist over time.

**Table 3:** Adjusted DiD^a^ estimates for the effect of coverage change on healthcare consumption in euros and quantity according to the years considered for comparison before and after the change

Insurance benefits	T0^b^		T1^c^		T2^d^		T3^e^		T4^f^	
	DiD	*p*	DiD	*p*	DiD	*p*	DiD	*p*	DiD	*p*
**In euros**										
Visits to GPs	−5.2	.468	−7.0	.355	4.3	.580	−12.5	.090	−.9	.906
Visits to specialists	10.9	.607	−16.9	.419	1.4	.944	−6.7	.743	10.9	.578
Pharmacy	−9.8	.697	.4	.989	18.2	.565	−9.1	.749	9.7	.748
Biological analyses	−1.7	.817	**34.9**	**.011**	13.4	.238	**33.2**	**.012**	11.2	.293
Paramedics	21.7	.562	24.8	.449	35.5	.260	48.6	.130	58.0	.059
Medical acts	22.8	.168	14.5	.470	−19.9	.298	**38.9**	**.037**	3.4	.846
Dental care^g^	**15.2**	**.026**	−8.6	.251	**−20.3**	**.003**	6.8	.306	−4.8	.413
Dental prostheses	76.0	.080	**320.9**	**<.001**	20.5	.723	**399.2**	**<.001**	99.0	.071
Vision	−8.4	.501	**52.9**	**<.001**	21.2	.148	**45.3**	**.001**	13.8	.285
Hospital	19.2	.803	−67.6	.446	−48.5	.624	−44.5	.578	−27.8	.752
Total	121.5	.306	**406.9**	**.005**	143.4	.328	**536.8**	**<.001**	**272.0**	**.046**
**In quantity**										
Visits to GPs	−.3	.342	−.2	.464	.2	.418	−.5	.090	−.1	.943
Visits to specialists	.3	.581	−.3	.596	.1	.855	−.1	.998	.3	.474
Pharmacy	−1.6	.530	.9	.747	1.6	.609	−.5	.871	.4	.897
Biological analyses	−.6	.413	.5	.574	1.1	.191	−.1	.827	.5	.450
Paramedics	2.4	.593	2.0	.618	2.9	.427	4.6	.239	5.4	.136
Medical acts	.4	.116	−.1	.962	**−.7**	**.022**	.4	.152	−.3	.336
Dental care^g^	.3	.096	−.1	.652	−.3	.090	.2	.240	−.1	.919
Dental prostheses	.2	.071	**.4**	**<.001**	−.1	.628	**.6**	**<.001**	.1	.238
Vision	−.1	.419	**.3**	**.004**	.2	.082	**.3**	**.014**	.1	.233
Hospital	.9	.359	−1.5	.200	−1.7	.191	−.5	.643	−.7	.560
Total	.7	.909	2.9	.635	4.3	.472	4.0	.505	5.4	.357

^a^Estimates were adjusted for all covariates listed in Table 2 except any dependent spouse; ^b^ T0: before = 2 years prior, after = 1 year prior; ^c^ T1: before = 1 year prior, after = 1 year after; ^d^ T2: before = 1 year prior, after = 2 years after; ^e^ T3: before = 2 years prior, after = 1 year after; ^f^ T4: before = 2 years prior, after = 2 years after; ^g^ Including dental consultations. A *p*-value less than 0.05 (in bold) indicates a significant effect. DiD: difference-in-differences. The monthly premium depends on the level of coverage that was subscribed to and the insuree’s age; the price starts at 27 € for BC and 33 € for EC [35]

Irrespective of the matching method used, the same order of magnitude and statistical significance were found for the DiD estimators. The only exception concerned significance when using the Kernel method, which was associated with a much greater statistical power, yielding an almost systematic significance of the DiD estimators, even though it was very small. Detailed results may be provided on request.

## Discussion

Our results are generally consistent with the literature on moral hazard in contract theory, according to which individuals may consume more if they are insured as they do not have to bear the full financial consequences of their healthcare consumption [38]. Indeed, we generally observed higher levels of consumption for the types of care that were better reimbursed with EC, except for hospital care, which is an exception typically observed in field studies [10, 23, 39–41]. In particular, the rise in consumption primarily concerned dental prostheses and vision, which are poorly reimbursed by CHI in general but have much improved coverage in EC than in BC. Nevertheless, in the case of the US, some papers revealed that the increase after coverage extension was due more to a quantity effect than a price effect [26, 28], which is not what we observed. In our study, a strong increase in expenses was observed in the first year after a change in coverage, whereas no significant effect in units was found in the 2 years following the change. This finding suggests that the most costly items of care (e.g., dental prostheses, glasses) were consumed first after the change. Similarly, we noticed a decrease in hospital consumption after the change in coverage, though not significant, that may indicate some substitution between hospital and community-based care, as has been suggested previously by Chandra et al. [42].

However, one should be cautious in drawing conclusions from these results about the presence of moral hazard for two main reasons. First, the French healthcare system is characterised by relative freedom for patients to choose how they access the healthcare system and which healthcare providers they consult [43], although a soft form of gate-keeping was introduced in 2004. We cannot, however, exclude that providers carry some of the responsibility for the increase in healthcare consumption [44], notably in the case of dental care, where the cost of interventions can be fixed relatively freely. Consequently, we cannot discard the presence of a small dose of supply-induced demand. Second, consistent with the findings of O’Malley et al. [45] and Manning et al. [46], our longitudinal data did not show any ratchet effect. The rise in consumption after the change in coverage was only temporary, especially for dental prostheses. This finding may indicate a catching-up behaviour following improvements in coverage, which is expected by the pent-up demand theory [27], and then a return to normalcy. However, in the 2 years preceding the increase in coverage, no decline in consumption was observed, which would mean that if there was a latent demand for healthcare, it would represent a long-term rather than a short-term need. Regarding the increase in consumption observed in the year preceding the change, it is possible that some part of the latent demand might have been expressed prior to the change. Before the change, some EC insurees may have put off the care they needed for as long as possible, but some of them were obliged to initiate their costly care even before the extension of their insurance coverage was effective. This situation may arise, for example, in the case of the development of a dental abscess, which requires urgent care, and the insuree has no other choice than to be treated immediately. In this case, the increase in care consumption would be expected to continue and intensify after the change in coverage. This was observed for dental prostheses and vision.

The preceding observations also show that the occurrence of a major health problem may represent a driver for subscribing to EC. The available data allow us to study factors upstream of the decision to extend health insurance coverage. The underlying rationality seems to be grounded on an objective assessment of the individual’s health status. It seems that insurees base their decisions on their actual rather than expected needs. This idea is suggested by the increase in the use of hospitals or dental prostheses just before changing healthcare coverage. The evolution of hospital care is all the more relevant in that people do not generally choose to go to hospital and that this is likely to reflect a change in the underlying health of the beneficiaries [47, 48]. Moreover, the strong price effect observed before the change in coverage suggests that some EC insurees seemed to enter a sequence of costly care before making a decision to extend their coverage. For example, the increase in the consumption of dental care or prostheses just before extension was accompanied by a sharp increase in the consumption of dental prostheses after the extension of coverage.

Finally, given the substantial differences in healthcare consumption observed between the two groups all over the period, one might think that the insurees choosing EC would form a specific population that is very different from the insurees remaining with BC. This is evidenced by Table 2 for observable characteristics such as gender, age, employment, health care needs, etc. These differences suggest the presence of selection among insurees. This self-selection effect, as insurees decide to opt for EC, may result in adverse selection for the insurer, as EC may attract individuals with poorer health as suggested by the increase in healthcare consumption prior to the change in coverage. However, these differences are taken into account by our empirical strategy, which allows for comparing insurees with similar characteristics and comparable trends in healthcare consumption before the change. Accounting for observable and unobservable differences between EC and BC insurees raises the question of the validity of our comparisons between groups and of our measures of changes over time and leads to the principal limits and strengths of our study.

Overall, our study presents two major strengths. The first is the analysis of the impact on healthcare consumption accompanying an actual major shift in health insurance coverage and not just some marginal changes to coverage, as may have been the case in several previous studies [32, 33]; this analysis is unlike other studies in which the exact nature of the change in coverage was not well known [49]. The second strength is the comprehensive documentation of healthcare consumption for a long time before and after the change in health insurance coverage, the actual guarantees of which are known. O’Malley et al. [45] noted the importance of healthcare consumption behaviour before the change in coverage for studying moral hazard. Moreover, our estimations highlight the influence of the choice of the time periods considered for such analysis with respect to the magnitude of the findings. However, the results from symmetric DiD, i.e., with the same number of periods before and after the treatment date as T4, should be the preferred estimations, as pretreatment outcomes are used to correct for selection bias [50].

Nonetheless, the interpretation of our findings must account for the two principal limitations of our data and related methodological concerns, which we tried to minimize using appropriate econometric techniques. First, the change in EC was decided by the insurees themselves and was not imposed on a randomly selected sample, which may result in a self-selection effect. The insurees choosing EC may be very different from the insurees remaining with BC from the beginning of the study period. In this regard, the self-selection of insurees for EC may contribute to adverse selection for the insurer. To avoid possible bias in the selection, we used matching techniques to compare EC insurees with paired BC insurees according to several major observable characteristics. Given the large number of controls, it was possible to identify a BC pair with identical characteristics (gender, age, marital status, affiliation of children, any hospital or specialised care) for each EC insuree. Moreover, the sensitivity analysis conducted on the matching procedures yielded the same results. We also used DiD models to control for time-invariant characteristics that were not observable, such as health risk, risk aversion or hypochondria, which may explain some of the differences in healthcare consumption between the two groups. Second, we did not have explicit information on the health status. Our results may thus be biased by differences in the underlying healthcare needs. The use of matching techniques may have solved a significant part of the problem, as it can be assumed that insurees who are comparable in gender and age present a similar level of health risk. In addition, our matching variables included the variables related to the presence of any previous hospital or specialised care that were considered as proxies for health status. In this respect, the gap between the BC curves before and after matching in Fig. 2 clearly indicates that an important part of the discrepancies in healthcare expenses between BC and EC insurees was taken into account with the control of observable characteristics. In addition, DiD models control for the general trend observed in healthcare consumption in both BC and EC groups and any external factor that may have an impact on it, for example, an especially virulent influenza epidemic. Moreover, DiD modelling may help account for the differences in health status between EC and BC insurees if the health status remained the same before and after the change in coverage. However, DiD modelling does not control for the individual changes in health status during the observation period. This is why we introduced explanatory variables in the DiD regression to control for observable variations in healthcare needs (hospital admissions and visits to specialists). Heckman’s two-step model could have been used to correct for self-selection for EC coverage, particularly that due to health. However, it would have been based on the same proxies for health status and would not have allowed for approaching the causal effect of a change in coverage on healthcare consumption as it is not dynamic.

## Conclusion

Health insurance coverage impacts healthcare consumption, especially in the case of costly care. This finding is timely and relevant to the current debate over the implementation of 100% reimbursement by compulsory NHI for glasses, dental treatment, and hearing aids in France [7]. It appears, however, that more research is needed to investigate medium- and longer-term effects of a move to more extensive health insurance coverage. As observed in our study, both catching-up behaviour and moral hazard are likely to play a role in the observed increase in healthcare consumption. It would be interesting to investigate the respective magnitude of the different effects involved, with more hindsight than the two years after the change because the observed increase may be mostly a result of meeting pent-up demand, as was observed in the OHIE [51]. Moreover, it is possible that further structural changes in the pattern of consumption will emerge with time, for example, shifts in recourse to different types of healthcare providers.

## Supplementary information

**Additional file 1.** Average healthcare consumption in euros during the years before and after the change.

**Additional file 2.** Average healthcare consumption units during the years before and after the change.

## Data Availability

The dataset analysed during the current study is not publicly available due to the protection of personal data according to the European Union’s General Data Protection Regulation.

## Abbreviations

BC: Basic coverage
CHI: Complementary health insurance
DiD: Difference-in-differences
EC: Extended coverage
EM: Exact matching
GP: General practitioner
HIE: Health insurance experiment
MGEN: Mutuelle Générale de l’Éducation Nationale
NHI: National health insurance
NHS: National health service
OHIE: Oregon health insurance experiment
PSM: Propensity score matching

## References

[1] Brook RH (1990). Quality of ambulatory care: epidemiology and comparison by insurance status and income. Med Care.

[2] Newhouse JP, The Insurance Experiment Group. Free for all? Lessons from the RAND Health Insurance Experiment. Cambridge, Mass.: Harvard University Press; 1993.

[3] Brook RH (1983). Does free care improve adults' health? Results from a randomized controlled trial. N Engl J Med.

[4] Keeler EB (1985). How free care reduced hypertension in the health insurance experiment. J Am Med Assoc.

[5] Bailit H (1985). Does more generous dental insurance coverage improve oral health?. J Am Dent Assoc..

[6] Keeler EB (1992). Effects of cost-sharing on the use of medical services and health. J Med Pr Manag.

[7] Casassus B (2017). Macron's vision for the French health system. Lancet..

[8] Manning WG (1987). Health insurance and the demand for medical care: evidence from a randomized experiment. Am Econ Rev.

[9] Finkelstein A (2012). The Oregon health insurance experiment: evidence from the first year. Q J Econ.

[10] Gerfin M, Schellhorn M (2006). Nonparametric bounds on the effect of deductibles in health care insurance on doctor visits - Swiss evidence. Health Econ.

[11] Boes S, Gerfin M (2016). Does full insurance increase the demand for health care?. Health Econ.

[12] Gerfin M (2015). Healthcare demand in the presence of discrete price changes. Health Econ.

[13] Nolan A (2017). Layte. The impact of transitions in insurance coverage on GP visiting among children in Ireland. Soc Sci Med.

[14] Lambregts TR, van Vliet RCJA (2018). The impact of copayments on mental healthcare utilization: a natural experiment. Eur J Health Econ.

[15] Magnussen Landsem M, Magnussem J (2018). The effect of copayments on the utilization of the GP service in Norway. Soc Sci Med.

[16] Olsen CB, Melberg HO (2018). Did adolescents in Norway respond to th elimination of copayments for general practitioner services. Health Econ.

[17] Ramos P, Almeida A (2016). The impact of an increase in user costs on the demand for emergency services: the case of Portuguese hospitals. Health Econ.

[18] Dickey H (2016). “Doctor my eyes”: a natural experiment on the demand for eye care services. Soc Sci Med.

[19] Puig-Junoy J (2015). Free medicines thanks to retirement: impact of coinsurance exemption on pharmaceutical expenditures and hospitalization offsets in a national health service. Health Econ.

[20] Garcia-Gomez P, Mora T, Puig-Junoy J (2018). Does €1 per prescription make a difference? Impact of a capped low-intensity pharmaceutical co-payment?. Appl Health Econ Health Policy.

[21] Jakobsson N, Svensson M (2016). Copayments and physicians visits: a panel data study of Swedish regions 2003-2012. Health Policy.

[22] Card D (2008). The impact of nearly universal insurance coverage on health care utilization: evidence from Medicare. Am Econ Rev.

[23] Gemmill M (2006). Insurance coverage and the heterogeneity of health and drug spending in the United States. Geneva Pap R I Iss P.

[24] Anderson GM (1991). A comparison of cost-sharing versus free care in children: effects on the demand for office-based medical care. Med Care.

[25] Grabowski DC, Gruber J (2007). Moral hazard in nursing home use. J Health Econ.

[26] Keeler EB, Rolph JE (1988). The demand for episodes of treatment in the health insurance experiment. J Health Econ.

[27] Long SH (1998). Do people shift their use of health services over time to take advantage of insurance?. J Health Econ.

[28] Keeler EB (1988). The demand for episodes of mental health services. J Health Econ.

[29] DREES (2018). Les dépenses de santé en 2017. Résultats des comptes de la santé. Édition 2018.

[30] Chevreul K (2015). France: health system review. Health Syst Transit.

[31] Buchmueller TC (2004). Access to physician services: does supplemental insurance matter? Evidence from France. Health Econ.

[32] Chiappori PA (1998). Moral hazard and the demand for physician services: first lessons from a French natural experiment. Eur Econ Rev.

[33] Franc C (2016). Supplemental health insurance and healthcare consumption – a dynamic approach to moral hazard. Health Econ.

[34] Grignon M (2008). Does free complementary health insurance help the poor to access health care? Evidence from France. Health Econ.

[35] MGEN. 2018. https://www.mgen.fr/offres-sante-prevoyance/trouvez-votre-offre-sante/. Accessed 27 Mar 2018.

[36] INSEE. 2017. http://www.insee.fr/. Accessed 30 Jan 2017.

[37] Éco-Santé. 2015. http://www.ecosante.fr/. Accessed 18 May 2015.

[38] Arrow JK. Uncertainty and the welfare economics of medical care. Am Econ Rev. 1963:941–73.

[39] Bolhaar J (2012). A dynamic analysis of the demand for health insurance and health care. Eur Econ Rev.

[40] Sapelli C, Vial B (2003). Self-selection and moral hazard in Chilean health insurance. J Health Econ.

[41] Shapiro MF (1986). Effects of cost sharing on seeking care for serious and minor symptoms. Results of a randomized controlled trial. Ann Intern Med.

[42] Chandra A (2010). Patient cost-sharing and hospitalization offsets in the elderly. Am Econ Rev.

[43] Rodwin VG (2003). The health care system under French national health insurance: lessons for health reform in the United States. Am J Public Health.

[44] Woodworth L, Romano PS, Holmes JF (2017). Does insurance status influence a patients’ hospital charge?. Appl Health Econ Health Policy..

[45] O'Malley JP (2016). Health care utilization rates after Oregon's 2008 Medicaid expansion: within-group and between-group differences over time among new, returning, and continuously insured enrolees. Med Care.

[46] Manning WG (1985). The demand for dental care: evidence from a randomized trial in health insurance. J Am Dent Assoc.

[47] Thygesen LC (2015). Potentially avoidable hospitalizations in five European countries in 2009 and time trends from 2002 to 2009 based on administrative data. Eur J Pub Health.

[48] Weeks WB (2015). Rates of admission for ambulatory care sensitive conditions in France in 2009–2010: trends, geographic variation, costs, and an international comparison. European J Health Econ.

[49] Dormont B, Péron M (2016). Does health insurance encourage the rise in medical prices? A test on balance billing in France. Health Econ.

[50] Chabé-Ferret S (2015). Analysis of the bias of matching and difference-in-difference under alternative earnings and selection processes. J Econ.

[51] Springer R (2018). Oregon Medicaid expenditures after the 2014 affordable care act Medicaid expansion: over-time differences among new, returning, and continuously insured enrolees. Med Care.

