# Supplementary material for: Healthcare consumption after a change in health insurance coverage: a French quasi-natural experiment
Source: Health Econ Rev. 2020 Jun 11;10:17. doi: 10.1186/s13561-020-00275-y (PMC7291705; doi:10.1186/s13561-020-00275-y)
Supplement: Supplementary file 1 — Additional file 1. Average healthcare consumption in euros during the years before and after the change. [file 13561_2020_275_MOESM1_ESM.docx]

| **Additional file 1** Average healthcare consumption in euros during the years before and after the change | | | | |
| --- | --- | --- | --- | --- |
| Insurance benefits^1^ | 2 years prior^2^ | 1 year prior | 1 year after | 2 years after |
| Visits to GPs |  |  |  |  |
| EC | 92.0 | 100.0 | 90.4 | 86.9 |
| BC before EM | 98.3 | 100.0 | 90.2 | 81.6 |
| BC after EM | 91.9 | 100.0 | 92.7 | 85.7 |
| Visits to specialists |  |  |  |  |
| EC | 74.5 | 100.0 | 79.4 | 71.4 |
| BC before EM | 98.8 | 100.0 | 82.6 | 82.6 |
| BC after EM | 95.8 | 100.0 | 94.4 | 87.5 |
| Pharmacy |  |  |  |  |
| EC | 89.4 | 100.0 | 99.7 | 95.3 |
| BC before EM | 99.7 | 100.0 | 96.6 | 95.6 |
| BC after EM | 93.0 | 100.0 | 98.3 | 96.7 |
| Biological analyses |  |  |  |  |
| EC | 78.4 | 100.0 | 125.7 | 97.7 |
| BC before EM | 90.8 | 100.0 | 104.2 | 103.8 |
| BC after EM | 80.9 | 100.0 | 108.3 | 95.3 |
| Paramedics |  |  |  |  |
| EC | 54.8 | 100.0 | 96.8 | 87.0 |
| BC before EM | 92.1 | 100.0 | 109.9 | 108.4 |
| BC after EM | 61.1 | 100.0 | 98.9 | 93.0 |
| Medical acts |  |  |  |  |
| EC | 67.5 | 100.0 | 100.6 | 85.0 |
| BC before EM | 94.3 | 100.0 | 102.6 | 109.6 |
| BC after EM | 75.8 | 100.0 | 102.3 | 255.2 |
| Dental care^3^ |  |  |  |  |
| EC | 69.4 | 100.0 | 85.4 | 63.0 |
| BC before EM | 105.8 | 100.0 | 255.8 | 233.3 |
| BC after EM | 86.6 | 100.0 | 86.4 | 78.2 |
| Dental prostheses |  |  |  |  |
| EC | 64.9 | 100.0 | 246.7 | 117.7 |
| BC before EM | 167.0 | 100.0 | 210.8 | 180.1 |
| BC after EM | 97.6 | 100.0 | 97.1 | 87.1 |
| Vision |  |  |  |  |
| EC | 77.1 | 100.0 | 127.4 | 109.6 |
| BC before EM | 95.7 | 100.0 | 84.5 | 86.6 |
| BC after EM | 71.7 | 100.0 | 77.0 | 87.1 |
| Hospital |  |  |  |  |
| EC | 51.1 | 100.0 | 59.2 | 82.0 |
| BC before EM | 98.7 | 100.0 | 99.9 | 101.0 |
| BC after EM | 89.3 | 100.0 | 95.3 | 184.7 |
| ^1^ As visits to osteopaths, orthodontics and maternity benefits concerned few insurees, the results for these three categories of care are not presented. ^2^ Index base 100 = 1 year prior. ^3^ Including dental consultations. EM: exact matching | | | | |
