# Supplementary material for: Healthcare consumption after a change in health insurance coverage: a French quasi-natural experiment
Source: Health Econ Rev. 2020 Jun 11;10:17. doi: 10.1186/s13561-020-00275-y (PMC7291705; doi:10.1186/s13561-020-00275-y)
Supplement: Supplementary file 2 — Additional file 2. Average healthcare consumption units during the years before and after the change. [file 13561_2020_275_MOESM2_ESM.docx]

| **Additional file 2** Average healthcare consumption units during the years before and after the change | | | | |
| --- | --- | --- | --- | --- |
| Insurance benefits^1^ | 2 years prior^2^ | 1 year prior | 1 year after | 2 years after |
| Visits to GPs |  |  |  |  |
| EC | 94.7 | 100.0 | 92.1 | 86.8 |
| BC before EM | 100.0 | 100.0 | 86.4 | 77.3 |
| BC after EM | 93.5 | 100.0 | 90.3 | 83.9 |
| Visits to specialists |  |  |  |  |
| EC | 74.3 | 100.0 | 80.0 | 68.6 |
| BC before EM | 100.0 | 100.0 | 85.7 | 85.7 |
| BC after EM | 95.0 | 100.0 | 95.0 | 85.0 |
| Pharmacy |  |  |  |  |
| EC | 78.0 | 100.0 | 125.1 | 147.1 |
| BC before EM | 94.2 | 100.0 | 105.8 | 113.9 |
| BC after EM | 75.5 | 100.0 | 126.9 | 157.2 |
| Biological analyses |  |  |  |  |
| EC | 71.8 | 100.0 | 92.3 | 94.9 |
| BC before EM | 113.6 | 100.0 | 100.0 | 100.0 |
| BC after EM | 73.3 | 100.0 | 113.3 | 126.7 |
| Paramedics |  |  |  |  |
| EC | 54.3 | 100.0 | 91.3 | 81.5 |
| BC before EM | 89.5 | 100.0 | 110.5 | 107.9 |
| BC after EM | 65.5 | 100.0 | 118.2 | 96.4 |
| Medical acts |  |  |  |  |
| EC | 77.1 | 100.0 | 91.7 | 77.1 |
| BC before EM | 94.7 | 100.0 | 100.0 | 94.7 |
| BC after EM | 84.8 | 100.0 | 90.9 | 93.9 |
| Dental care^3^ |  |  |  |  |
| EC | 73.3 | 100.0 | 93.3 | 73.3 |
| BC before EM | 133.3 | 100.0 | 300.0 | 300.0 |
| BC after EM | 100.0 | 100.0 | 100.0 | 88.9 |
| Dental prostheses |  |  |  |  |
| EC | 83.3 | 100.0 | 200.0 | 100.0 |
| BC before EM | 200.0 | 100.0 | 200.0 | 200.0 |
| BC after EM | 133.3 | 100.0 | 100.0 | 100.0 |
| Vision |  |  |  |  |
| EC | 77.8 | 100.0 | 122.2 | 111.1 |
| BC before EM | 100.0 | 100.0 | 83.3 | 83.3 |
| BC after EM | 85.7 | 100.0 | 100.0 | 100.0 |
| Hospital |  |  |  |  |
| EC | 60.0 | 100.0 | 77.5 | 92.5 |
| BC before EM | 91.3 | 100.0 | 104.3 | 100.0 |
| BC after EM | 68.6 | 100.0 | 100.0 | 142.9 |
| ^1^ As visits to osteopaths, orthodontics and maternity benefits concerned few insurees, the results for these three categories of care are not presented. ^2^ Index base 100 = 1 year prior. ^3^ Including dental consultations. EM: exact matching | | | | |
